# Spatial proteomics in precision medicine: technologies, bioinformatics, and translational applications

**DOI:** 10.1093/pcmedi/pbaf040

**Published:** 2026-01-08

**Authors:** Yiwen Li, Yusheng Zhang, Ying Zhang, Qing Wang, Boyang Ji, Hongjun Yang, Xianyu Li

**Affiliations:** Beijing Key Laboratory of Traditional Chinese Medicine Basic Research on Prevention and Treatment for Major Diseases, Experimental Research Center, China Academy of Chinese Medical Sciences, Beijing 100700, China; Beijing Key Laboratory of Traditional Chinese Medicine Basic Research on Prevention and Treatment for Major Diseases, Experimental Research Center, China Academy of Chinese Medical Sciences, Beijing 100700, China; Beijing Key Laboratory of Traditional Chinese Medicine Basic Research on Prevention and Treatment for Major Diseases, Experimental Research Center, China Academy of Chinese Medical Sciences, Beijing 100700, China; School of Life Sciences, Beijing University of Chinese Medicine, Beijing 100029, China; Bioinnovation Insitute, Copenhagen 2200, Denmark; Beijing Key Laboratory of Traditional Chinese Medicine Basic Research on Prevention and Treatment for Major Diseases, Experimental Research Center, China Academy of Chinese Medical Sciences, Beijing 100700, China; China Academy of Chinese Medical Sciences, Beijing 100700, China; Beijing Key Laboratory of Traditional Chinese Medicine Basic Research on Prevention and Treatment for Major Diseases, Experimental Research Center, China Academy of Chinese Medical Sciences, Beijing 100700, China

**Keywords:** spatial proteomics, multi-omics, mass spectrometry, imaging, precision medicine

## Abstract

Protein function is inherently spatial: the same molecule can elicit distinct biological outcomes depending on its localization, interacting partners, and surrounding microenvironment. Spatial proteomics enables systematic *in situ* characterization of protein localization, abundance, and interactions across subcellular to tissue scales, surpassing the resolution and contextual information accessible to conventional bulk proteomics. Recent technological advances including DNA-barcoded multiplexing methods, cyclic fluorescence platforms, and mass spectrometry imaging have substantially increased multiplexing capacity, sensitivity, and spatial accuracy. These capabilities directly support clinically relevant applications, such as tumor immune microenvironment analysis, mapping of protein aggregation in neurodegeneration, growth factor dynamics during tissue repair, patient stratification, pharmacodynamic mapping, and target-engagement assessment. Computational innovations, including graph neural networks, self-supervised embeddings, and workflow management tools (e.g. Snakemake, Nextflow), further enhance cell segmentation, noise reduction, and multi-modal data integration, enabling extraction of robust, spatially resolved proteomic information from complex tissues. Future research will aim to standardize protocols, enable real-time clinical analysis, and develop 3D spatial proteome maps to advance spatial proteomics toward precision diagnostics and targeted therapies.

## Introduction

Protein function is inherently spatial: the same molecule can trigger distinct biological outcomes depending on its localization, interacting partners, and surrounding microenvironment. Spatial proteomics (SP) has emerged as a transformative approach to map protein distribution within native tissue contexts, overcoming the spatial information loss of conventional lysate-based proteomics [1]. Recent advances have enabled subcellular resolution, whole-tissue profiling, and dynamic pharmacodynamic monitoring [2], supporting key applications such as tumor immune microenvironment analysis, protein aggregation mapping in neurodegeneration, and growth factor dynamics in tissue repair.

**Figure 1:**
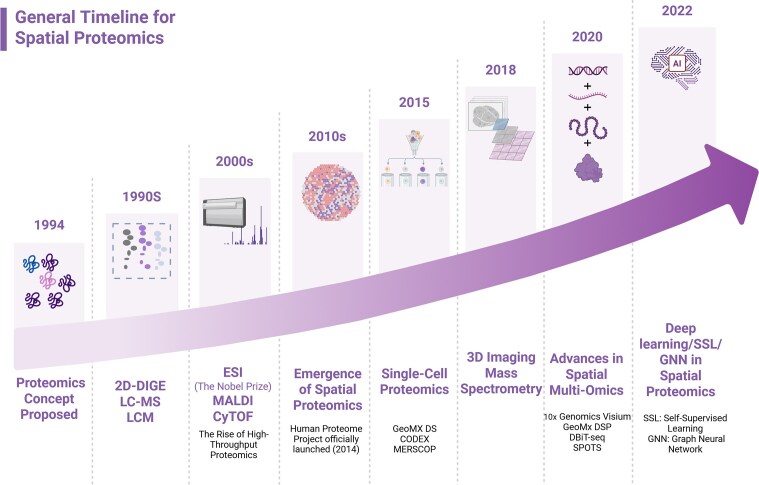
Recent artificial intelligence advances in spatial proteomics.The timeline summarizes representative methodological milestones from early proteomics and imaging innovations to current spatial multi-omics and high-capacity, representation-learning approaches; classical machine-learning methods are not shown. Abbreviations: 2D-DIGE, two-dimensional difference gel electrophoresis; LC–MS, liquid chromatography–mass spectrometry; LCM, laser capture microdissection; ESI, electrospray ionization; MALDI, matrix-assisted laser desorption/ionization; CyTOF, cytometry by time-of-flight; DSP, Digital Spatial Profiler; CODEX, CO-Detection by indEXing; DBiT-seq, deterministic barcoding in tissue sequencing; SPOTS, Spatial PrOtein and Transcriptome Sequencing; AI, artificial intelligence; SSL, self-supervised learning; GNN, graph neural network.

For clinicians and biomedical investigators, understanding the strengths and limitations of these emerging SP approaches will be essential for selecting appropriate platforms, interpreting results, and translating spatial information into meaningful biological or therapeutic insights. With ongoing methodological innovation (Fig. 1), SP has achieved increasing resolution [3–5] and is progressively integrating with multi-omics and computational approaches to refine disease classification and inform therapeutic decisions [6]. This review highlights the clinical relevance and application scenarios of SP in disease stratification and precision interventions, while minimizing technical detail. Looking ahead, its potential in digital pathology, spatially guided drug delivery, and personalized treatment planning is expected to further advance precision medicine.

## Technological advances in SP

Driven by application needs, the technological advancement of SP aims to achieve: higher spatial resolution to interrogate protein expression at the subcellular scale; enhanced sensitivity and coverage to explore low-abundance, cell-type-specific key proteins; and increased throughput and scalability to adapt to diverse sample types while reducing costs and improving efficiency.

**Figure 2:**
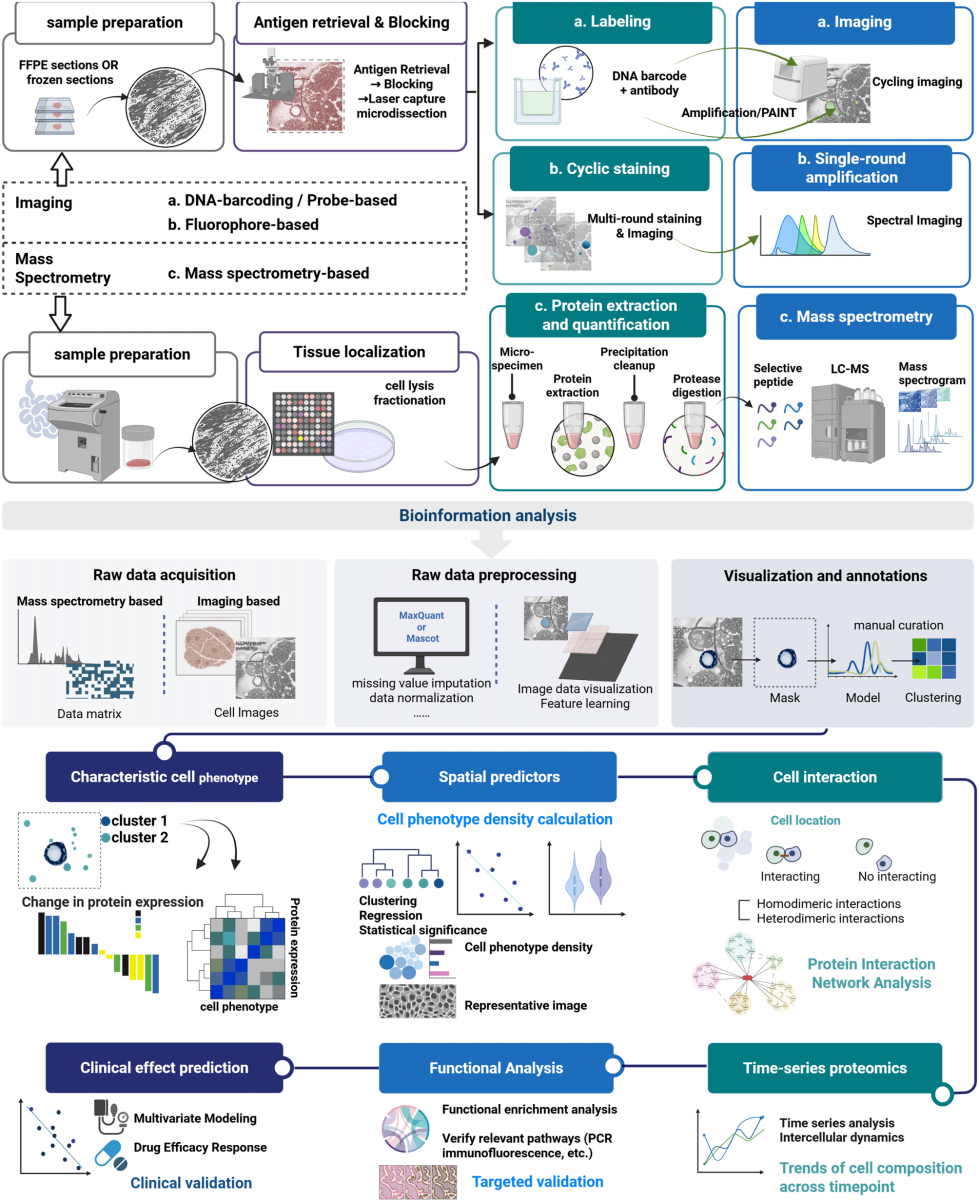
SP analysis workflow and interpretable clinical questions.

Accordingly, we focus this review on two major categories: imaging-based platforms (Fig. 2), which preserve histological context while achieving high multiplexity, and mass spectrometry (MS)-based approaches (Fig. 2), which enable high spatial resolution, exceptional sensitivity, and high throughput for unbiased protein discovery with distinct trade-offs in resolution and throughput (Table 1). Broadly defined, SP also encompasses conventional histopathological techniques such as H&E staining and immunohistochemistry (IHC) [7], which preserve tissue architecture and enable extraction of spatially resolved protein-associated information. Distinct technologies exhibit specific application profiles (Table 2). Concurrently, we will outline parallel advances in sample preparation that underpin these platforms. This transition elevates SP investigations from the discovery of individual proteins to the identification of statistically significant biomarkers.

**Table 1:** SP platforms, methods, and sample workflows.

Module/theme	Representative technologies/platforms	Primary applications	Key advantages	Limitations/considerations	Ref.
Imaging-based					
Cyclic fluorescence	t-CyCIF IBEX, MELC, cellDIVE, TSA, COMET™	>50-plex whole-slide immunophenotyping, enhanced pathology readouts	Compatible with standard fluorescence microscopes, relatively low barrier for clinical translation	Limited by achievable cycle number (typically ∼10–20 cycles, corresponding to ∼30–60-plex panels in routine practice, with occasional reports >60-plex); spectral crosstalk and epitope-dependent staining quality; substantial image-registration burden across rounds, especially for whole-slide scans	[8–14]
Signal amplification	Opal	Low-to-mid-plex target analysis, enhanced sensitivity and signal-to-noise ratio	Single-round imaging for speed; effective for low-abundance targets	Typically supports ∼4–8-plex protein panels per slide (6–7 Opal dyes plus nuclear counterstain); requires careful panel design to avoid bleed-through and epitope competition; dependence on well-validated epitopes; potential compression of dynamic range for very abundant targets	[15, 16]
DNA barcoding/probe-based	PhenoCycler/CODEX, Immuno-SABER, MACSima/MICS, DNA Exchange Imaging	High-plex tissue microenvironment and cellular interaction mapping	Ultrahigh multiplexing, decouples multiplexity from optical limits, subcellular resolution	Strong antibody/epitope dependence, with validated research panels usually in the ∼30–60-marker range (some systems reporting ∼50–100 targets); ≥10–20 hybridization/imaging cycles increase cumulative photobleaching and probe degradation; quantitative non-linearity complicates absolute protein copy-number estimation	[17–25]
Super-resolution imaging	DNA-PAINT/SUM-PAINT	Nanoscale protein localization and interaction mapping	Single-molecule resolution, exceptional spatial precision	Very low throughput (typical fields of view limited to tens–hundreds of cells with acquisition times of ≥1–3 h per region); multiplex panels usually in the dozens of targets rather than hundreds; demands highly stable samples and specialized optics with active drift correction	[26, 27]
Mass spectrometry-based					
Mass cytometry imaging	MIBI-TOF, IMC, FAXP, DISCO-MS	High-dimensional immune profiling, deep analysis of FFPE archives	Many simultaneous channels, low background, robust for archived tissues	High instrument and consumable cost; acquisition typically limited to ∼30–40 simultaneously imaged metal-tagged markers at ∼1 µm pixel size, so increasing area or resolution rapidly lengthens run times (often hours per slide for multi-mm² coverage); inherent trade-off between spatial resolution and practical throughput	[20, 28–35]
Elemental/isotopic imaging	LA-ICP-MS, NanoSIMS	Tissue mapping via elemental/isotopic tags	Provides orthogonal chemical specificity	Niche instrumentation and specialist operation; high-resolution modes (≤1–5 µm pixel size) are restricted to relatively small regions of interest (typically ≤mm²) with long acquisition times; many applications require pre-labeling with isotopic or elemental tags to achieve sufficient molecular specificity	[36–38]
Label-free MS imaging	MALDI-MSI, DESI, MALDI-IHC	Untargeted protein/metabolite discovery, drug distribution, PTM imaging	Broad molecular coverage, label-free, direct detection of modifications	Fundamental trade-off between spatial resolution and molecular depth: commercial MALDI-MSI systems typically operate at ∼10–50 µm pixel sizes to balance coverage and sensitivity; whole-section images can involve 10^4^–10^6^ pixels and require hours of acquisition; ion suppression and multi-step sample preparation (cryosectioning, matrix coating, washing) complicate robust quantification	[30, 39–43]
Deep proteomics	LOPIT/hyperLOPIT, nanoPOTS	Subcellular organelle mapping, single/few-cell proteomics from trace samples	Deep proteome coverage, high sensitivity, achieves subcellular context	Low analytical throughput: deep subcellular or single/few-cell LC-MS/MS runs typically take ≥1–2 h per gradient and are currently practical for at most tens to low hundreds of samples per study; workflows rely on extensive fractionation or highly specialized low-loss platforms (e.g. nanoPOTS chips), which are challenging to standardize at scale	[44–48]
Sample preparation and analysis strategies					
Tissue clearing	SHIELD, SWITCH	Volumetric imaging and proteomics of large tissues/whole organs	Preserves morphology, enables organ-scale coverage	Clearing and immunolabeling of whole organs usually require multi-day workflows (on the order of ∼2–7 days from fixation to imaging, depending on tissue size and protocol); may alter antigenicity and epitope accessibility for some antibodies; clearing chemistries and electrophoretic steps can introduce extraction bias for more labile proteins or lipids	[49–53]
Targeted region enrichment	IHC-LCM + DIA	Deep proteomic analysis of specific pathological regions	Enables *in situ* targeting, FFPE-compatible, suitable for low-abundance targets	Very low throughput: manual or semi-automated laser capture typically yields at most tens to a few hundred regions of interest per day, each comprising roughly 10²–10^4^ cells; labor-intensive microdissection and low protein input impose quantitative variability and limit routine application to small, pre-selected areas	[54]
Tissue stabilization	Protein-stabilizing chemistries, hydrogel embedding	Improved protein recovery and MS detection sensitivity, compatible with multiplex imaging	Enhances proteome preservation and MS signal	Protocols add multiple additional processing steps (often ≥1 extra day for embedding, polymerization, clearing/washes) and are not yet fully standardized across tissue types; differences in crosslinking and digestion conditions can introduce batch-specific shifts in peptide recovery and quantitative bias across experiments	[55, 56]
Computational analysis and AI	SpatialData, GraphCompass; Snakemake/Nextflow; GNN, TransGCN	Spatial statistics, cell–cell neighborhood mapping, scalable reproducible workflows	Standardized data frameworks; explicit modeling of spatial context	Model outputs and feature attributions can be difficult to interpret biologically; robust training typically requires on the order of 10^4^–10⁵ manually or semi-automatically annotated cells/spots per tissue and careful cross-validation; cross-platform variability in resolution, panel composition, and staining protocols leads to domain-shift issues that limit direct model transfer between datasets	[57–62]
Multi-omics integration	Spatial-CITE-seq, DBiT-seq, MUON, inClust+, spaVAE, wcSOP-MS	Protein–RNA–chromatin or protein–metabolite integration; FFPE-compatible voxel proteomics	True multimodal synergy; near-cellular resolution	Marked data-scale mismatch across modalities (e.g. transcriptome-wide vs ∼20–100-plex protein vs sparse metabolite panels) and limited same-cell multimodality (often <10–20% of profiled cells have truly joint measurements) increase modeling complexity; joint analysis of multi-slide, multi-omics datasets can demand >100 GB RAM and GPU-class compute, constraining routine use to well-resourced center	[63–68]

**Table 2:** Comparative performance of SP technologies.

Feature	DNA-barcoding	Fluorophore-based	MS-based
Plex capacity	Highest (50–100)	Moderate (20–60)	Low (no marker panel)
Proteome coverage	Moderate	Moderate	Highest
Spatial resolution	Subcellular	Optical-limit	5–50 μm
Morphology	Excellent	Excellent	Limited
Quantification	Semi-quantitative	Semi-quantitative	Calibratable quantitative
PTM detection	No	No	Yes
Clinical readiness	Medium	Highest	Low
Cost/complexity	High	Moderate	High

### DNA-barcoded antibody/probe–based spatial profiling

DNA-barcoding approaches couple protein-binding antibodies to unique oligonucleotide tags and recover spatial protein identity through iterative hybridization or orthogonal readouts, thereby decoupling multiplexing capacity from optical channels and enabling subcellular high-plex maps [19, 28, 69, 70]. This approach transcends the physical limitations of optical channels, achieving a high-degree of multiplexing (50–100+ markers) unattainable with conventional optical methods, which represents its core advantage. Two implement action families dominate: cyclic hybridization, which builds tens to >50-plex whole-slide images by repeated hybridize–image–strip cycles [18, 19, 71–73], and DNA-based amplification/transient-binding schemes (e.g. Immuno-SABER, DNA-PAINT) as well as adaptations that couple barcodes to non-fluorescent detection modalities [20, 21, 26, 27]. Cyclic systems retain histomorphology but face concrete limitations—antibody/epitope dependence, cumulative photobleaching and probe degradation [74], rigid and non-rigid tissue shifts that require fiducials or advanced registration [75], and quantitative nonlinearity from hybridization kinetics and steric effects [76]. Such systematic biases lead to the underestimation of protein complexes, thereby compromising biological interpretation. Such biases also hinder the comparison and integration of results across studies. Amplification/PAINT variants increase sensitivity or spatial precision but typically trade throughput for longer acquisition times and add variability linked to concatemer heterogeneity or buffer-dependent kinetics [20, 21, 77]. Barcode strategies have also been ported to imaging mass cytometry (IMC) / multiplexed ion beam imaging (MIBI) and sequencing-based platforms [63, 64], expanding analytic depth at the cost of different sample-preparation, instrumentation, and cross-modal integration challenges [78, 79].

For clinical translation, best practices emphasize systematic antibody quality control (QC), robust image registration, cross-platform validation with MS/IMC [20, 28], and computational pipelines that mitigate batch effects and nonspecific signals while generating interpretable per-cell metrics [11, 39, 40, 72, 80–83]. Combined with spatial transcriptomics (e.g. Visium) [84], their integration enables mutual validation and complementary insights, thereby enabling functional analysis of proteins even at single-cell or low-abundance levels.

### Fluorophore-based

Fluorophore-based multiplexed imaging remains the modality most seamlessly aligned with routine clinical pathology, as it preserves conventional optical workflows and maintains high-fidelity tissue morphology compatible with H&E and standard IHC. These platforms use either cyclic antibody staining (sequential stain–image–bleach or strip cycles) or single-round amplification/spectral separation to increase parameter counts while retaining optical histology. Cyclic imaging methods offer superior multiplexing capacity, whereas single-round approaches provide greater throughput but are difficult to scale beyond ∼10 markers without advanced spectral unmixing. Cyclic methods (e.g. t-CyCIF and IBEX) [9, 85, 86] achieve tens to >50 markers with whole-slide compatibility but are constrained by cycle number, imaging time, and inter-round registration requirements [9, 76, 87]. Single-round amplification such as TSA/Opal reduces cycles and speeds acquisition but demands careful panel design and spectral unmixing to avoid bleed-through [81]. These issues are particularly pronounced in FFPE tissues, where autofluorescence and stromal heterogeneity amplify spectral overlap and can generate false co-expression signals if not rigorously corrected [13]. Hyperspectral imaging improves fluorophore separation and autofluorescence removal at the cost of greater instrument complexity and data processing burden [81]. To support analytical robustness and enable eventual clinical translation, mitigation strategies are now emphasized across three levels. At the antibody level, per-antibody QC, validation across fixation conditions, and optimized dye/panel configuration reduce variability. At the imaging level, the use of fiducials, non-rigid registration, and standardized exposure/bleaching controls minimize inter-round drift. At the computational level, robust spectral unmixing, batch correction, and cross-platform validation are increasingly regarded as essential to achieve reproducibility across instruments, tissues, and laboratories [82].

Both H&E/IHC slides and high-plex fluorescence images can be analyzed using artificial intelligence (AI) to quantitatively decode spatial patterns in tissue architecture, cellular morphology, and protein expression [88–90]. This approach enables the discovery of prognostic and predictive features that are not discernible by conventional visual inspection (Table 3). For instance, nuclear morphometric and tissue architectural features extracted from H&E and IHC slides can be leveraged to investigate heterogeneity within the cancer immune microenvironment and cellular responses to therapy [7, 91].

**Table 3:** Application of imaging and MS-based SP.

Approach	Method^a^	Application	Ref.
**Imaging**			
DNA labeled	PhenoCycler (formerly CODEX)	Mouse spleen Human bone marrow niche	[19, 103]
	GeoMx DSP	Melanoma and NSCLC	[4, 104]
	CosMx		[5]
	Immuno-SABER	Melanoma	[20, 21]
	Spatial-CITE-seq		[105]
Fluorophore labeled	PhenoCycler-Fusion	Head and neck cancer-specific tumors	[3, 24]
	CyCIF	Melanoma Colorectal cancer tumors	[14, 105, 106]
	TRIPODD		[85]
	SUM-PAINT	Neuron and synapse	[26]
	CLASP	Suborganelle proteome and membrane protein topology	[107]
Metal labeled	MIBI-TOF	Invasive breast cancer Lymph nodes, tonsils, and cancer tissues	[28, 29, 33]
	IMC	Breast cancer, liver cancer, NSCLC, brain tumor, ovarian cancer	[35, 57, 108–112]
	ACE	Polycystic kidney	[113]
**Mass spectrometry**			
	LOPIT hyperLOPIT	Mouse pluripotent stem cells	[44, 114]
	DISCO-MS	Brian in Alzheimer’s disease mice Atherosclerosis in humans .	[99]
	TransitID	Cancer	[115]
	nanoPOTS	Neuron in amyotrophic lateral sclerosis Islet tissue from patients with type 1 diabetes mellitus	[45, 46, 48]
	DVP	Invasive melanoma	[116]
	PCP	Liver	[117, 118]

^a^PhenoCycler (formerly CODEX, Co-Detection by indexing), GeoMx DSP (GeoMx Digital Spatial Profiler), CosMx (CosMx Spatial Molecular Imager), Immuno-SABER (Immuno-Simultaneous Amplification and Biodetection of RNA), Spatial-CITE-seq (Spatially-resolved transcriptomics with Cellular Indexing of Transcriptomes and Epitopes by sequencing), PhenoCycler-Fusion (PhenoCycler Fusion, a platform for highly multiplexed imaging), CyCIF (Cyclical Immunofluorescence), TRIPODD (Tissue-wide Rapid Imaging of Proteins with Optical Density Detection), SUM-PAINT (Super-resolution Ultrastructure Mapping by Photoactivation and In situ Nanocluster Tomography), CLASP (Click chemistry-based Labeling And Subsequent Proximity-dependent labeling), MIBI-TOF (Multiplexed Ion Beam Imaging-Time of Flight), IMC (Imaging Mass Cytometry), ACE (Atomic Cartography of Elements), LOPIT (Localization of Organelle Proteins by Isotope Tagging), hyperLOPIT (High-resolution LOPIT), DISCO-MS (DISsection COupled to Mass Spectrometry), TransitID (Translational Initiation Dynamics Identified by DIA), nanoPOTS (Nanodroplet Processing in One pot for Trace Samples), DVP (Direct Visualization Proteomics), PCP (Protein Correlation Profiles), NSCLC (Non-Small Cell Lung Cancer).

### MS-based

MS-based SP directly measures endogenous peptides or intact proteins *in situ* (label-free or targeted), offering unbiased proteome discovery and the ability to detect post-translational modifications that are inaccessible to antibody panels [92, 93]. Its core advantage lies in the ability to identify proteins not covered by antibody panels and to detect protein functionalities such as post-translational modifications, capabilities that are unattainable with other methods. Major mass spectrometry imaging (MSI) modalities include matrix-assisted laser desorption/ionization-MSI (MALDI-MSI) and desorption electrospray ionization (DESI) for label-free molecular imaging, and laser-ablation approaches [36] for element/isotope mapping; these methods provide broad molecular coverage and high chemical specificity [31] but traditionally trade spatial resolution and sample throughput for depth of detection. Advances in matrices, ion sources, and instrument sensitivity [94] have substantially increased ion yields and lowered background, improving detectability of low-abundance species. Microfluidics-assisted and microdissection coupling strategies further push spatial granularity toward near-cellular (∼25 μm) resolution and permit integration of transfer-learning workflows for complex tissues [95]. Nevertheless, MS-based approaches face persistent challenges for routine clinical application: complex sample preparation, ion suppression and matrix effects that complicate quantification; limited spatial resolution relative to high-plex imaging; and sensitivity limits intrinsic to single-cell proteomics that require carrier strategies or signal amplification to reach robust proteome coverage. Consequently, MS methods are particularly powerful for mechanism-oriented and discovery studies [96], while complementary imaging-based platforms remain preferable when high spatial resolution and direct histomorphology are prioritized. Its advantageous applications include uncovering novel disease mechanisms and assessing pharmacokinetics [97, 98]. In studies of tumor heterogeneity, protein aggregation in neurodegenerative diseases, and spatial drug distribution, MSI serves as the premier tool for the unbiased discovery of new biomarkers and mechanisms of action. Furthermore, in biomarker validation, it provides a list of candidate targets for the subsequent development of targeted antibody imaging panels (Table 3).

### Sample preparation and tissue clearing

Efficient sample preparation and tissue clearing are essential for maximizing the performance of spatial proteomics, as fixation, extraction, and clearing protocols directly influence protein recovery, epitope accessibility, spatial integrity, and ultimately the reliability of downstream multiplexed imaging or MS-based readouts.

Recent strategies such as DISCO-MS integrate solvent-based tissue clearing with MS workflows [99]. By removing lipids and rendering tissues transparent, these methods enable deep proteome coverage from intact organs, facilitating unbiased discovery of molecular mechanisms in neuroscience and systemic disease models.

Protein-preserving chemical stabilization methods [52, 53] protect proteins against harsh clearing conditions, allowing high-fidelity antibody labeling and iterative imaging in large tissues or even whole organs. Enrichment-based approaches [54] combine IHC with on-slide proteomic digestion to improve sensitivity for low-abundance targets *in situ*. These innovations expand tissue size and molecular depth accessible to SP, but they also introduce technical trade-offs: clearing agents can alter antigenicity; solvent exposure may cause protein loss; and long processing times reduce throughput for translational pipelines. To mitigate these effects, optimized buffer chemistries, standardized digestion protocols, and cross-validation with conventional IHC or bulk MS are increasingly recommended [27, 70].

Overall, optimized sample preparation and tissue clearing provide a critical foundation for high-quality SP, enabling the integration of multiplexed imaging with deep proteomic readouts [31, 41, 100–102]. They are applicable across multiple SP platforms, and are particularly important when working with clinically relevant specimens, such as FFPE biopsies, organ slices, etc.

## Bioinformatics and multi-omics synergy

### AI in SP

Bioinformatics transforms spatial images and spectra into biologically and clinically actionable insights. Modern SP datasets pose substantial analytical challenges that exceed the capacity of classical image processing or per-marker statistics. AI models address these challenges by improving segmentation, denoising, spatial context modeling, and cross-modal integration.

Before biological modeling becomes feasible, raw images and spectra must be transformed into spatially organized molecular features. Contemporary workflows integrate spatial-aware data structures, image preprocessing, feature extraction, and statistical modeling into reproducible pipelines. Popular ecosystems [58, 62, 119] support multi-format data ingestion, spatial statistics, neighborhood analysis, and visualization. Deep generative models produce modality-aligned representations [120, 121], enabling denoising, imputation of missing channels, and harmonization across staining batches or imaging platforms. Workflow managers [122] enforce reproducibility and scalable execution across compute environments. These components collectively provide the foundation upon which AI methods can operate.

For dimensionality reduction, clustering, and visualization, classical methods remain useful for exploratory analysis, but modern spatial problems often benefit from machine-learning models that explicitly model spatial context or cross-modal relationships. Recent AI advances applied to SP include deep generative models and self-supervised embeddings that improve data integration and denoising across modalities, graph neural networks that formalize cell–cell interaction modelling on tissue graphs [59], and task-specific networks for cell-type calling or phenotype prediction that borrow ideas from single-cell transcriptomics and imaging-based pathology [123, 124].

AI approaches have improved segmentation and signal de-noising, and enabled the learning of cross-modal mappings to integrate imaging and sequencing readouts. However, several limitations deserve emphasis. Model interpretability and clinical explainability remain limited; deep models can be brittle to staining, fixation, or instrument shifts and demand substantial annotated training data for robust generalization. Standardization gaps (per-marker QC, metadata reporting, and benchmark datasets) further hinder cross-study reproducibility and regulatory acceptance. To advance translational readiness, we recommend combining (i) rigorous per-marker QC and small-scale orthogonal validation [109], (ii) use of transfer-learning/domain-adaptation strategies to reduce annotation burden [64], (iii) adoption of containerized, workflow-managed pipelines for reproducibility, and (iv) community benchmark datasets and challenge-driven evaluation to identify and characterize failure modes and to evaluate calibration methods [60].

In practice, successful spatial proteomics projects synergistically combine classical statistical methods with a targeted AI component. These analyses should be implemented within containerized, workflow-managed pipelines [61] that enforce provenance tracking, scalability, and reproducibility. Moving forward, emphasizing transparent model reporting, open dataset sharing, and the adoption of standardized metrics will be essential to translate SP discoveries into reliable, clinically deployable applications.

### Multi-omics synergy in clinical challenges

**Figure 3:**
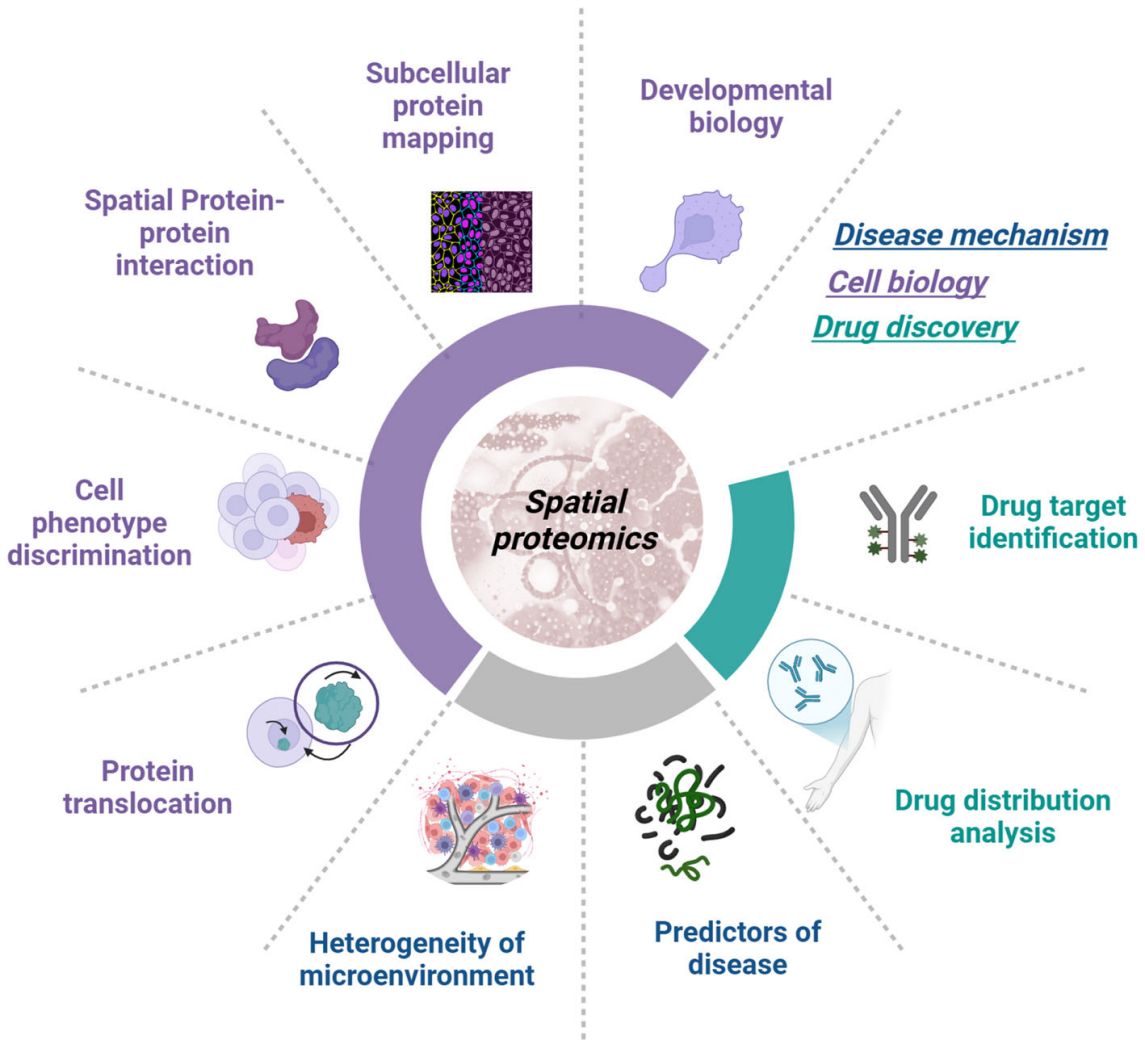
Technical issues of integrating spatial multi-omics.

The complexity of biological systems necessitates integrating SP with complementary omics technologies and computational frameworks to achieve clinically actionable insights (Fig. 3) [125]. Recent advancements in high-resolution spatial mapping [95] combine microfluidics-based imaging and deep learning to enable proteome-wide profiling. Similarly, wcSOP-MS integrates laser capture microdissection with surfactant-assisted processing to quantify ∼900–4 600 proteins in tissue voxels as small as 1–100 cells, making it compatible with clinical FFPE samples for region-specific pathway discovery [65]. These technologies bridge the gap between bulk tissue analysis and single-cell resolution, uncovering spatially restricted molecular niches critical for disease mechanisms.

Dynamic profiling techniques further enhance functional annotation. The oblique line scan imaging technique achieves nanoscale spatial precision and sub-millisecond temporal resolution, tracking protein motion and transient interactions in living cells—capabilities vital for drug screening and dissecting signaling dysregulation [126]. Cross-link assisted spatial proteomics (CLASP) extends these insights to subcellular compartments, resolving mitochondrial membrane proteomes and synaptic vesicle heterogeneity. CLASP identified mislocalized disease-associated proteins in neurodegenerative disorders, offering targets for organelle-specific therapies [107].

Spatial multi-omics is costly, but simultaneous measurements from the same sample are possible, integrating spatial transcriptomics and proteomics [127], or spatial metabolomics with proteomics [128]. Challenges include standardizing sample preparation, ensuring antibody specificity, and managing computational demands. Multimodal data fusion frameworks address the challenges of integrating disparate omics layers. True multi-omics experiments that measure multiple modalities in the same cells remain rare [66]. The performance of cell segmentation algorithms is influenced by tissue type and disease state, and the opportunity for parallel measurements of the same cells across multiple modalities remains limited. inClust+ [68], a deep generative model, was developed for single-cell and spatial transcriptomics and supports cross-modal imputation and batch correction. It can integrate paired scRNA-seq and MERFISH data and has been extended to tri-modal datasets (gene expression, chromatin accessibility, and protein abundance), enabling label transfer and imputing missing protein abundance from CITE-seq references. spaVAE and its extensions [121], jointly modeling spatial transcriptomics and ATAC-seq, resolving immune-stromal crosstalk in tumor subtypes, and predicting immunotherapy-responsive niches [11]. Platforms like PhenoCycler-Fusion and DBiT-seq further enable concurrent mRNA–protein detection, revealing microvasculature-pigmented epithelium interactions in early organogenesis [67]. Taking the integration of SP and spatial transcriptomics as an example, these technologies exhibit differences in resolution and sensitivity, leading to issues such as inconsistent data formats and varying signal intensities, which complicates cross-omics data analysis. Specifically, SP often struggles with low-abundance protein detection, whereas spatial transcriptomics generates high-sensitivity spatial expression maps of transcripts but with lower spatial resolution. These discrepancies make direct comparison and integration challenging. To address these issues, the PhenoCycler-Fusion platform has been used for high-throughput transcriptomic mapping of tumors and their microenvironments, demonstrating that interactions between CD8+ cells and tumors or other immune cells are associated with positive responses to immunotherapy [24]. Meanwhile, the soScope toolkit enhances the analytical capabilities of spatial omics data by comprehensively evaluating and improving various spatial omics technologies [129, 130].

To address these challenges, researchers are exploring unified data processing frameworks and advanced algorithms. For instance, mathematical models are used to standardize data signals from different omics, ensuring they can be compared on the same scale. Machine learning and deep-learning techniques, such as spaVAE, have been applied in spatial transcriptomics to explore the spatial characteristics of gene expression, offering insights that are also applicable to SP [121]. Hierarchical integration strategies leverage SP for initial exploration to identify key proteins and their spatial distributions. Findings are then validated and enriched using scRNA-seq and ATAC sequencing (scATAC-seq), improving accuracy and offering deeper insights into cellular heterogeneity and function [123].

Technologically, methods such as spatial-CITE-seq and spatial-ATAC-RNA-seq [63] enable the simultaneous capture of protein expression and transcript information within cells, achieving critical multimodal data integration that supports spatial multi-omics. Other platforms, such as SeqFISH+, enhance protein detection by combining antibody specificity with DNA barcode detectability [131], while SM-Omics [132] and SPOTS [133] facilitate concurrent detection of RNA and proteins, enabling effective analysis of multiple biomolecules. Despite the current focus on transcriptome studies [134], joint analyses of SP and transcriptomics are becoming more common, aided by advanced frameworks under development for multimodal data integration [68, 135].

Spatial multi-omics integration offers additional dimensions of information, enabling a panoramic view of cellular and tissue organization [136]. For instance, integrating spatial metabolomics and proteomics has allowed researchers to perform single-cell segmentation [137], phenotypic analysis, and submicron-level metabolite analysis, uncovering novel cell-type-specific metabolic states in endometrial and lung cancers. By combining samples collected at different time points with multi-layered omics data, researchers can elucidate dynamic changes in biological processes such as development or disease progression, advancing disease research and precision medicine [138].

## Translational applications of SP in diagnosis

SP offers unprecedented molecular and architectural resolution, but its translational utility critically depends on a multi-step analytical pipeline—where variation at each stage can alter downstream clinical conclusions (Fig. 2). Differences in instrumentation, data formats, and preprocessing steps such as peak extraction [139], peptide identification via database matching [140], spatial quantification, etc. [141], introduce platform-specific biases and batch effects that must be carefully controlled. Because no single workflow is universally applicable, current SP pipelines are often highly customized, being optimized for tissue type, analytical depth, or multiplexing requirements.

These technical choices have direct consequences for biological and clinical interpretation. Robust spatial statistics and neighborhood modeling, together with appropriate visualization strategies, are essential for accurately resolving protein aggregation, cellular interactions, and disease-specific microenvironments [142, 143]. In addition, spatial localization reference resources [96, 144–146] and time-series analysis workflows [147–150] should be standardized across studies, as discrepancies in these components can introduce substantial variability into downstream biological interpretations. Ultimately, SP addresses the following three pivotal questions in therapeutic development. What target proteins exist within the tissue microenvironment? Where and how do therapeutic interventions act? Does the drug successfully reach and engage its intended target? (Table 4).

**Table 4:** SP in precision medicine.

Category	Platform/method	Representative cases	Key strengths	Limitations/cautions	Ref.
**Imaging-based**	PhenoCycler (formerly CODEX)	Whole-slide high-plex immune/tumor microenvironment (TME) mapping in mouse spleen and human marrow; breast HER2 heterogeneity; head and neck tumor profiling	Subcellular resolution; preserved histology; scalable multiplexing	Antibody/epitope dependence, with validated panels typically ∼40–60 markers per run (some platforms reporting ∼100+ in optimized settings); requires ∼10–20 fluidic/imaging cycles per slide, which accumulate photobleaching and cross-cycle registration errors and introduce quantitative nonlinearity	[19, 24, 72, 75, 82, 103]
	Immuno-SABER	Amplified multiplex imaging in melanoma and other tumors	Higher sensitivity via DNA concatemers; reduced false negatives	Longer acquisition times for amplified channels (often tens of minutes to hours per large field of view); concatemer-length variability and buffer-dependent hybridization kinetics require tight optimization; practical panel sizes in current tissue studies are usually in the ∼10–20-target range	[20, 21]
	DNA-PAINT/SUM-PAINT	Nanoscale mapping of neuronal synapses; single-protein-level spatial proteomics	Ultra-high precision; single-molecule readouts	Severe throughput and time constraints—3D nanoscale datasets typically cover only tens–hundreds of cells with ≥ 1–3 h acquisition per region; although barcoding allows in-principle unlimited multiplexing, practical panels are usually restricted to tens of targets; requires highly drift-stable optics and often microfluidics	[26, 27, 77]
	t-CyCIF/IBEX (cyclic IF)	>50-plex whole-slide immunophenotyping;	Compatible with standard microscopes; wide adoption	Iterative staining/imaging (commonly ∼10–20 cycles to reach ∼30–60-plex datasets) increases photobleaching and tissue distortion; 3–4 fluorophores per cycle introduce spectral bleed-through; whole-slide registration and quality control across cycles add substantial computational and workflow burden	[9, 14, 76, 106]
	MIBI-TOF/IMC (metal-labeled)	High-dimensional immune profiling in breast cancer; archival tissues; early atherosclerosis cell phenotyping	Low background; many channels; FFPE-friendly	High capital and consumable cost; typical panels of ∼30–40 metal-tagged antibodies with ∼0.5–1 µm pixel size mean that imaging multi-mm² regions often requires hours of beam/laser time; increasing resolution or area further reduces practical throughput	[28, 29, 57, 108, 109, 151]
	ACE/element cartography	Element/isotope-linked tissue mapping	Orthogonal chemical specificity	Depends on access to lightsheet- or similar large-volume imaging plus high-end compute; teravoxel-scale datasets (multi-TB per cleared brain) demand specialized storage and GPU resources, and atlas registration/cluster-wise statistics can take hours per experiment; pipeline complexity limits routine deployment outside expert centers.	[82]
**MS-based/sample**	MALDI/DESI-MSI	Label-free spatial proteome/metabolome for diagnosis and drug mapping (e.g. thyroid nodule classification; target engagement)	Broad molecular coverage; PTM/peptide level; chemical specificity	Fundamental trade-off between spatial resolution and depth—routine MALDI-MSI operates at ∼10–50 µm pixels to keep sensitivity and run time manageable; whole-section scans can contain 10^4^–10^6^ pixels and require hours of acquisition; ion suppression and multi-step sample preparation (sectioning, matrix/spray, washes) complicate robust quantification	[39, 40, 43, 92, 93]
	LOPIT/hyperLOPIT	Organelle-level protein localization; subcellular relocalization under disease or diet	Proteome-wide compartment maps	Requires extensive subcellular fractionation and multiplexed labeling; deep organelle-resolved maps typically need dozens of LC-MS/MS runs per experiment and many hours to days of instrument time, so routine use is largely limited to tens–low hundreds of samples/conditions per study	[44, 114, 117]
	nanoPOTS/single-cell MS	Single-/few-cell proteomes in neuro/diabetes tissues; DVP for archival skin	High sensitivity from trace samples	Single-/few-cell workflows use long LC gradients (30–120 min per run), yielding on the order of ∼10–30 cells per day on standard platforms; carrier/boost and TMT multiplexing improve sensitivity but add complexity and potential batch effects, so throughput remains far below single-cell transcriptomic methods	[45, 46, 48, 116]
	Microfluidics-assisted high-res SP	Near-cellular voxels with transfer learning in complex tissues	Improved spatial granularity; ML integration	Microfluidic voxelization plus downstream LC-MS/MS generates near-cellular grids but at the cost of complex chip fabrication and handling; current implementations process at most tens–hundreds of voxels per sample with substantial computational burden for transfer-learning and model training, limiting adoption beyond specialized labs	[65, 95]
	DISCO-MS & tissue clearing	Deep proteome from intact organs (neuro, systemic disease) with morphology preserved	Whole-organ coverage; discovery-grade depth	Tissue clearing and labeling of intact organs typically require multi-day protocols (often ∼3–7 days from fixation to MS-ready samples); clearing chemistries can alter antigenicity and extraction efficiency for certain protein classes; integration with downstream MS/imaging pipelines remains technically demanding and not yet standardized	[99]
	IHC-LCM + DIA	Region-specific, FFPE-compatible pathway discovery	Targets low-abundance proteins *in situ*	Laser-capture microdissection is inherently low throughput—usually yielding at most tens to low hundreds of ROIs per day, each containing ∼10²–10^4^ cells; very low protein input from FFPE/IHC tissue makes absolute quantification and cross-sample normalization challenging and increases sensitivity to technical variation	[54]

### Advancing disease stratification beyond conventional taxonomy

**Figure 4:**
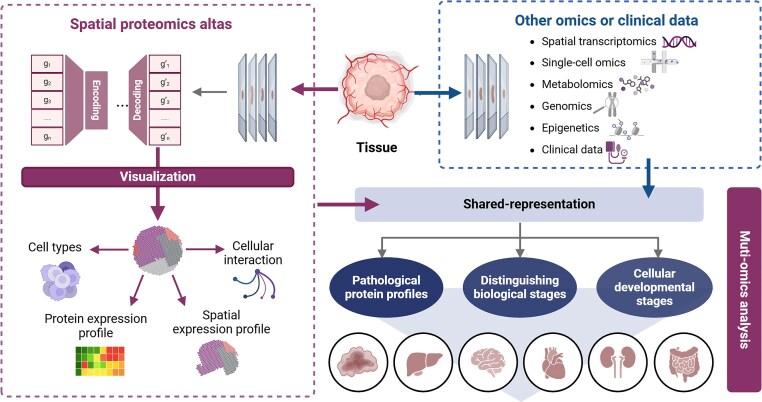
Applications of SP in discovering disease mechanisms and studying cellular functions.

Traditional classification based on histology, genetic mutations, or single markers cannot fully capture spatial heterogeneity of tissue microenvironments. SP enables multidimensional stratification by decoding protein networks, spatial gradients, and cellular niches beyond static morphology [152] (Fig. 4).

In oncology, SP resolves the spatial organization of protein expression within the tumor microenvironment (TME). It not only detects the presence of specific oncoproteins, immune checkpoints, and markers of cancer-associated fibroblasts [80, 153–155], but crucially maps their spatial gradients and co-localization patterns. This reveals functionally distinct malignant–immune niches and stromal architectures that underpin immune evasion and tumor-progression information largely lost in bulk tissue analysis. The direct clinical translation of this spatial mapping is evident in the automated, protein-signature-based classification of thyroid nodules by MALDI-MSI, achieving high diagnostic accuracy (100% sensitivity, 96% specificity) in a manner that complements traditional cytology [43]. In glioma, spatial multi-omics integration (transcriptomics, proteomics, computational modeling) delineates a five-layered cellular architecture organized by hypoxia-driven metabolic gradients [127]. This architecture transcends histologic classification, emphasizing microenvironmental regulation of tumor organization. Multiplexed imaging (e.g. MALDI-HiPLEX-IHC) further resolves molecular networks by simultaneously mapping 12 protein markers in complex pathologies [30].

In cardiovascular diseases, PhenoCycler identifies CD68+ vascular smooth muscle cells and foam cells as key mediators of atherosclerotic plaque instability [151], while near-infrared photoacoustic imaging (NIRAPA) links macrophage markers (CD74, CD163) to plaque vulnerability [156]. This spatial context directly links specific cellular proteomic states to clinical risk, offering a path toward imaging-based biomarkers for predicting stroke or myocardial infarction risk.

In Alzheimer’s disease, SP demonstrates that Aβ deposition reshapes hippocampal proteomes and modulates tau pathology [157]. Comparative analyses with primary age-related tauopathy blur conventional subtype boundaries [9]. Nanoscale proteomic profiling (e.g. nanoPOTS) further resolves neuronal alterations associated with TDP-43 inclusion formation, enabling stage-specific molecular definitions [48]. These insights uncover spatially resolved mechanisms of disease propagation and cellular vulnerability, paving the way for developing location-specific biomarker panels and targeted therapies aimed at halting pathological spread.

By bridging histomorphology with dynamic spatial proteomic maps, SP provides mechanism-driven disease stratification across oncology, cardiovascular, and neurodegenerative disorders. It enables a shift from population-based averages to spatially informed precision medicine, where diagnosis, prognosis, and therapeutic strategies are guided by the functional architecture of the tissue microenvironment [127, 158, 159].

### Progression risk identification: spatiotemporal early warning

Disease recurrence is closely related to time, but at the same time, the spatial characteristics of ‘latent foci that have not been cleared’ or ‘areas of immunosuppression failure’ in the disease microenvironment are also important factors. Therefore, SP is helpful to predict disease recurrence.

In oncology, SP has been applied to noninvasively stratify patients and predict prognosis in highly heterogeneous tumors. For example, the integration of ambient ionization MS (SpiderMass) with AI has enabled real-time classification and risk assessment in glioblastoma, a cancer characterized by complex spatial variability [160]. Beyond mere molecular abundance, spatial localization of key metabolites—such as lactate—has been shown to influence protein post-translational modifications and transcription factor activity (e.g. p53), revealing previously underappreciated regulatory layers in metabolic–signaling networks [158]. Through the integration of multi-omics data (including transcriptomics) and subsequent targeted validation, researchers are able to unravel the precise features and underlying mechanisms of tumor recurrence and progression. Multi-omics spatial profiling in bladder cancer, for instance, correlates chromosomal instability, transcriptomic subtypes, and immune infiltration with high chromosomal instability associated with poorer outcomes [154, 161–164]. In breast cancer, SP reveals extracellular matrix proteomic heterogeneity during ductal carcinoma *in situ* to invasive breast cancer transition [165]. Similarly, in cervical squamous cell carcinoma (CSCC), SP captures chemotherapy-induced immune signaling transitions (MP6 to MP7 via IFN-γ), which predict immunotherapy responsiveness [136].

SP also reveals pathological remodeling in cardiovascular and metabolic diseases. In abdominal aortic aneurysm, CRP-associated inflammatory cell localization predicts disease advancement [166], while in atherosclerosis, spatial transcriptomics and imaging link macrophage markers (CD74, CD163) to plaque vulnerability, offering predictive value for clinical management [156]. In the liver, nutrient gradient-driven mitochondrial heterogeneity governed by AMPK/mTOR signaling [167] and dynamic mitochondria–lipid droplet interactions under metabolic stress [168] reflect spatially regulated adaptations with potential prognostic implications.

In hematologic malignancies, such as multiple myeloma, spatial multi-omics profiling has identified chromatin remodeling complexes (e.g. ARID1A) as spatially restricted drivers of disease progression via the IRF4 and MYC oncogenic axes [169].

Collectively, these studies demonstrate that SP is not only instrumental in refining disease stratification but also enables the identification of spatial biomarkers and molecular events predictive of progression and therapeutic outcomes.

### Discovery of microenvironment-specific therapeutic targets

SP has revolutionized the ability to decipher disease microenvironments by elucidating cellular distributions and functional post-translational modifications [170]. The latest SP technology significantly lowers the barrier for widespread adoption and innovation in SP research [9]. For example, spatial glycomics analyses using MALDI-MSI and CE-ESI-MS have shown that immunogenic motifs α-Gal and Neu5Gc exhibit organ- and region-specific distributions, highlighting potential spatial targets to reduce xenograft immunogenicity and optimize interventions [171]. When combined with proteomics, techniques like P2L enable detailed profiling of protein microenvironments associated with specific glycans, revealing how tumor cell-surface interactions are altered under immune pressure [92]. Multiplexed SP platforms are redefining our ability to dissect the TME; for instance, using MACSima™ imaging cyclic staining (MICS), researchers have developed a 121-marker immunophenotyping panel to profile immune cell subsets in hepatocellular carcinoma, unveiling immunosuppressive cellular neighborhoods in which tumor-associated myeloid cells interact with PD1 (high) exhausted T cells [25]. Deep visual proteomics applied to archived skin tissues from toxic epidermal necrolysis (TEN) patients has mapped >5 000 proteins at single-cell resolution, revealing strong activation of the JAK/STAT and interferon pathways that drive disease pathology [47]. SP further demonstrates its utility across diverse clinical contexts: it outperforms existing methods in annotating islet pathobiology in the Human Pancreas Analysis Program (HPAP) [172].

Protein kinase r-like endoplasmic reticulum kinase (PERK) inhibition enhances β cell immune tolerance via PD-L1 stabilization—offering a strategy to delay type 1 diabetes onset [173]. In neural tissues, single-molecule imaging of neuronal synapses with SUM-PAINT has quantified heterogeneous distributions of synaptic proteins and identified novel subtypes (e.g. GluA2/PSD95-rich synapses) that enhance our understanding of neural circuit diversity [27]. Using integrated scRNA-seq and PHENOCYCLER imaging, researchers revealed distinct spatial neighborhoods for hematopoietic stem and progenitor cells, including arterio-endosteal and adipocytic zones [174]. In AML samples, mesenchymal stromal cells were expanded and spatially co-localized with leukemic blasts, suggesting niche remodeling in disease [174]. Single-cell deep visual proteomics has demonstrated a strong spatial correlation between the proteome and the anatomical structure of the liver, identifying E-cadherin and Glul as markers of the portal vein and central vein, respectively [123]. In skin tissue research, spatial quantitative proteomics combined with laser capture microdissection and MS constructed a layered skin proteome map. This study revealed that TGFβ-induced protein (TGFBI) in the basement membrane regulates the growth of epidermal stem cells and wound healing, providing critical insights into skin homeostasis and wound repair [175]. When integrated with other omics technologies such as transcriptomics and metabolomics, SP offers a more comprehensive understanding of cellular and tissue functions. The distribution of lipid-associated macrophages and Kupffer cells in the liver, along with their corresponding microenvironmental circuits, confirmed the essential role of ALK1-BMP9/10 in liver homeostasis [176]. In the retina, combining multiprotein maps (4i), single-cell RNA-seq, and single-cell ATAC-seq uncovered spatial protein characteristics and spatiotemporal gene regulatory networks during retinal development [129]. Using spatial imaging combined with single-cell transcriptomics and proteomics, researchers identified unique molecular features and structural connections of cranial bone marrow in immune responses, providing new potential targets for the diagnosis, monitoring, and treatment of brain diseases [168, 177].

## Drug discovery and pharmacological mechanism

### Precision in therapeutic response prediction

**Figure 5:**
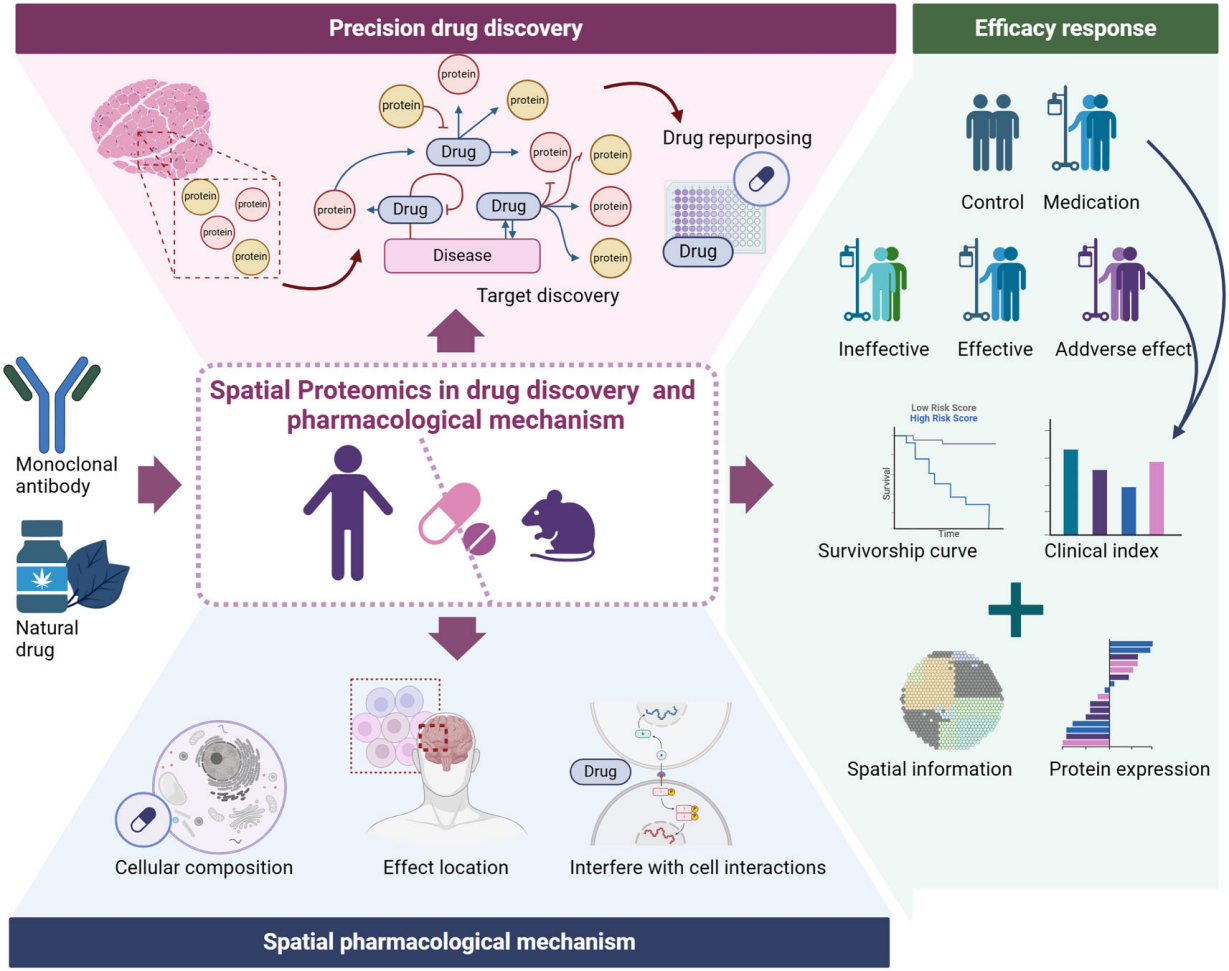
SP in drug discovery and pharmacological mechanisms.

The heterogeneous response to antibody-based therapies and small molecules underscores the critical need to map spatially restricted therapeutic resistance niches within the TME [160]. SP resolves adaptive signaling cascades driving therapy resistance (Fig. 5). In cutaneous squamous cell carcinoma (CSCC), chemotherapy-induced IFNγ-dependent transitions (MP6-to-MP7 states) predict immunotherapy responsiveness, guiding post-relapse therapeutic strategies [136]. Cutaneous T-cell lymphoma studies using Phenocycler identify spatially restricted biomarkers that differentiate pembrolizumab responders from non-responders, independent of PD-L1 expression [178, 179]. Mature TLS, enriched in responders, correlate with prolonged survival, highlighting their role as spatial determinants of immune checkpoint efficacy [179].

Beyond oncology, SP informs synergistic therapeutic design. In multiple myeloma, SWI/SNF chromatin remodelers sustain IRF4/MYC oncogenic networks, even in IMiD-resistant cases. Targeting SWI/SNF activity with SMARCA2/4 inhibitors disrupts these networks and synergizes with MEK inhibition, offering a paradigm for microenvironment-targeted combination therapies [169]. 3D pathology and agent-based modeling further simulate TME evolution under therapeutic pressure, predicting optimal drug sequencing to overcome resistance [180, 181].

Genetic, physiological, and environmental factors often lead to variable patient responses to the same treatment, even under identical drug-exposure conditions [182, 183]. For example, protein expression in a single cell line can vary significantly depending on drug interventions [184]. By integrating SP with single-cell and multi-omics approaches, researchers can uncover drug–target interaction mechanisms, predict drug pathways, and construct spatial networks of drug action, thereby advancing precision medicine [123, 185]. Advanced imaging technologies and computational methods further enhance SP’s capabilities, enabling precise tissue region analysis. Techniques such as color standardization, image registration, and deep-learning-based segmentation improve data reproducibility and accuracy, facilitating high-precision cellular and tissue segmentation across diverse imaging platforms [33, 186]. These advancements lay the groundwork for biomarker discovery in the TME and support the identification of functional cellular neighborhoods, which are critical for understanding drug–target interactions [54].

SP changes the treatment concept from a static biomarker assessment to a dynamic one by identifying specific immune and metabolic regions and linking signaling to clinical outcomes [57].

### Pharmacological mechanism analysis

SP holds advantages in constructing 3D pharmacological models [99] and tailoring personalized prescriptions [187] (Fig. 4). By observing the spatial proteomic response to drug interventions, it can enhance the benefits of individualized treatment. For instance, researchers identified local signal transducer and activator of transcription 1 (STAT1) phosphorylation activation as a key molecular mechanism in TEN, leading to the targeted use of the pan-janus kinase (JAK) inhibitor tofacitinib for TEN treatments [47]. The integration of SP with spatial transcriptomics and pathway analysis methods reveals how different cell types within the TME influence immune therapy responses [130]. The spatial distribution patterns of tumor cells are closely associated with patient prognosis, and SP can identify therapy-resistant cell populations post-treatment [188]. In breast cancer, combining SMAC mimetics with endocrine therapy enhances T cell migration and MHC-I-specific cytotoxicity by modulating NF-κB signaling, offering a novel treatment strategy [189].

Beyond oncology, SP provides valuable insights into the spatial distribution of known and potential pathways, adding a new dimension to precision therapy. In mouse pancreatic islets treated with PERK inhibitors, surface PD-L1 expression on β-cells was significantly upregulated. PERK inhibitors enhance immune tolerance by altering the immune function of β-cells, offering a new therapeutic target for type 1 diabetes [173]. SP has also revealed that drugs regulating the AMPK/mTOR pathways alter mitochondrial phenotypes in different regions, uncovering the link between mitochondrial heterogeneity and nutrient gradients in the liver [167]. Together, these findings demonstrate how SP, combined with single-cell and multi-omics technologies, provides new perspectives on drug–target interaction mechanisms. This approach deepens our understanding of cellular spatial relationships, microenvironmental changes, and biomarker identification in various cancers and immune diseases. In the research of traditional Chinese medicine and natural compounds, puerarin targets the gamma-aminobutyric acid type a receptor (GABAA) α1 subunit to inhibit dorsal motor nucleus of the vagus nerve activity and reduce intestinal fat absorption, establishing a new paradigm for natural compounds as precise modulators of single key targets in metabolic regulation and therapeutic innovation [190]. Using MALDI-TOF-MS and MALDI-MSI, the spatial distribution of α-Gal- and Neu5Gc-modified glycans in mammalian organs was systematically analyzed, providing a crucial foundation for addressing carbohydrate antigen-induced immune rejection in xenotransplantation [171].

## Discussion

SP has emerged as the “Method of the Year 2024” [1], revolutionizing drug discovery through spatiotemporal mapping of protein interactions that bridge pathological phenotypes with site-specific signaling pathways. By resolving low-abundance proteins in subcellular niches, SP identifies biomarkers for early diagnosis, therapy monitoring, and relapse prediction. In personalized treatment, SP enables precise drug and dosage selection by leveraging individual proteomic profiles. However, challenges such as limited sample sizes, rapidly evolving methodologies, and a lack of standardized protocols hinder data interpretation and introduce biases in understanding disease mechanisms and drug efficacy.

High-throughput data analysis in SP requires advanced computational resources and bioinformatics tools, often relying on manual input from experts. Future advancements should focus on enhancing high-resolution technologies, automating sample preparation and data analysis, and integrating multi-omics datasets. Standardized experimental and data processing protocols are critical for ensuring data comparability and reproducibility.

Despite challenges like cross-platform discrepancies, AI model interpretability [95], and high-dimensional data demands, SP shows great promise. Integrating high-resolution technologies, organoid models, AI, and spatial multi-omics will enable 3D and spatiotemporal proteomic mapping, expediting drug discovery and personalized treatments. The combination of SP with spatial transcriptomics will provide deeper insights into cellular communication, while innovations like specific digital twins and virtual clinical trials can reduce costs and risks, advancing both research and clinical applications in precision medicine.
